# Boldine, an Alkaloid from *Peumus boldus* Molina, Induces Endothelium-Dependent Vasodilation in the Perfused Rat Kidney: Involvement of Nitric Oxide and Small-Conductance Ca^2+^-Activated K^+^ Channel

**DOI:** 10.1155/2022/4560607

**Published:** 2022-02-16

**Authors:** Priscila de Souza, Rita de Cássia Vilhena da Silva, Luisa Mota da Silva, Viviane Miranda Bispo Steimbach, Karyne Garcia Tafarelo Moreno, Arquimedes Gasparotto Junior

**Affiliations:** ^1^Graduate Program in Pharmaceutical Sciences, Nucleus of Chemical-Pharmaceutical Investigations (NIQFAR), University of Vale do Itajaí, Itajaí, SC, Brazil; ^2^Laboratory of Electrophysiology and Cardiovascular Pharmacology, Faculty of Health Sciences, Universidade Federal da Grande Dourados, Dourados, Mato Grosso do Sul, Brazil

## Abstract

Boldine, 2,9-dihydroxy-1,10-dimethoxyaporphine, is the main alkaloid found in the leaves and bark of *Peumus boldus* Molina. In recent years, boldine has demonstrated several pharmacological properties that benefit endothelial function, blood pressure control, and reduce damage in kidney diseases. However, the renal vasodilator effects and mechanisms remain unknown. Herein, perfused rat kidneys were used to study the ability of boldine to induce vasodilation of renal arteries. For that, left kidney preparations with and without functional endothelium were contracted with phenylephrine and received 10–300 nmol boldine injections. The preparations were then perfused for 15 min with phenylephrine plus L-NAME, indomethacin, KCl, tetraethylammonium, glibenclamide, apamin, charybdotoxin, or iberiotoxin. In 30, 100, and 300 nmol doses, boldine induced a dose-and endothelium-dependent relaxing effect on the renal vascular bed. No vasodilator effects were observed in preparations lacking functional endothelium. While the inhibition of the cyclooxygenase enzyme through the addition of indomethacin did not cause any change in the vasodilating action of boldine, the nonselective nitric oxide synthase inhibitor L-NAME fully precluded the vasodilatory action of boldine at all doses tested. The perfusion with KCl or tetraethylammonium (nonselective K^+^ channels blocker) also abolished the vasodilatory effect of boldine, indicating the participation of K^+^ channels in the renal action of boldine. The perfusion with glibenclamide (selective ATP-sensitive K^+^ channels blocker), iberiotoxin (selective high-conductance Ca^2+^-activated K^+^ channel blocker), and charybdotoxin (selective high- and intermediate-conductance Ca^2+^-activated K^+^ channel blocker) did not modify the vasodilatory action of boldine. On the other hand, the perfusion with apamin (selective small-conductance Ca^2+^-activated K^+^ channel blocker) completely prevented the vasodilatory action of boldine at all doses tested. Together, the present study showed the renal vasodilatory properties of boldine, an effect dependent on the generation of nitric oxide and the opening of a small-conductance Ca^2+^-activated K^+^ channel.

## 1. Introduction

*Peumus boldus* Molina, popularly known as “boldo” or “boldu”, is a tree species belonging to the Monimiaceae family and native to central and southern Chile, where it occurs abundantly. In addition to popular use for the treatment of several ailments, widely known for gastric and liver disorders, boldo-based preparations are described in several official pharmacognostic texts, such as official pharmacopeias in Brazil, Chile, Germany, Portugal, Romania, Spain, and Switzerland [1, 2].

Boldo's active principles have been described in essential oils (ascaridol, cineol, esters, aldehydes, ketones, and hydrocarbons), alkaloids (boldine, isoboldine, and others), glycosides, and others (flavonoids, citric acid, gum, sugars, tannins, minerals, lipids, etc.). The barks are richer in alkaloids [3]. Most of the pharmacological studies of boldo describe the activities observed for the alkaloid boldine, defined as the main component of boldo tea [4]. The concentration of alkaloids in boldo leaves is estimated at 0.4%, and the concentration of boldine can reach more than 12% [5].

Indeed, a wide range of biological effects has been attributed to boldine. Boldine has been shown to possess antioxidant activity and anti-inflammatory effects, which is why it is often studied for oxidative stress-associated diseases. For instance, the protective effect of boldine on oxidative mitochondrial damage in streptozotocin-induced diabetic rats has been described [6], and the reduction on oxidative stress to induce its gastroprotective properties [7]. As for the pharmacological action on the cardiovascular and renal systems to control hypertension and pathologies such as diabetes, studies have shown the action of boldine in improving the endothelial function in diabetic mice through the inhibition of vascular oxidative stress and endothelial dysfunction [8]. In addition, studies have demonstrated that boldine treatment exerts endothelial protective effects in spontaneously hypertensive rats through the inhibition of NADPH-mediated superoxide production [9]. Boldine improved kidney damage in renovascular hypertension induced by the Goldblatt two-kidney one-clip model [10] and showed a renoprotective action in streptozotocin-induced diabetes in rats [11].

Despite the widespread use of the alkaloid boldine, its mechanism of action on renal function remains unclear. Considering the need for new approaches to the treatment of hypertension or contributing to the renoprotective actions against hypertension, this study aimed to investigate boldine's vasodilatory action by using an isolated and perfused rat kidney model to verify the hypothesis that boldine causes the direct relaxation of renal arteries. The mechanisms responsible for renal vasodilator actions were also explored.

## 2. Materials and Methods

### 2.1. Drugs

Boldine (≥98% purity) was purchased from Sigma-Aldrich (St. Louis, MO, USA). Heparin was obtained from Cristália, (São Paulo, SP, Brazil). Xylazine and ketamine hydrochloride were obtained from Vetec (Vetec, Duque de Caxias, RJ, Brazil). Acetylcholine chloride, apamin, charybdotoxin, dextrose, glibenclamide, iberiotoxin, indomethacin, tetraethylammonium chloride (TEA), N*ω*-nitro-L-arginine methyl ester (L-NAME), phenylephrine, sodium deoxycholate, NaCl, NaHCO_3_, KCl, CaCl_2_, MgSO_4_, KH_2_PO_4_, and ethylenediaminetetraacetic acid (EDTA) were purchased from Sigma-Aldrich (St. Louis, MO, USA).

### 2.2. Animals

Male Wistar rats, 3 months old, were obtained from the animal facility of the University of Vale do Itajaí (UNIVALI). The animals were kept at controlled room temperature (22 ± 2°C), 12-hour light/dark cycle, with free access to water and chow. All methodologies and procedures used here were submitted and approved by the Ethics Committee on Animal Use of UNIVALI, under authorization number 022/19p and followed all the National Control Council of Animal Experimentation recommendations.

### 2.3. Isolation and Perfusion of the Rat Kidney

**Figure 1:**
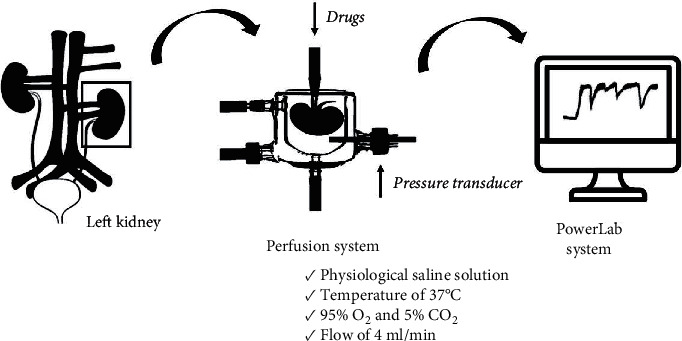
Diagram of the isolated perfused kidney experimental setup.

To avoid the appearance of clots in the renal vascular bed during the kidney isolation and perfusion procedure, the animals were previously treated with heparin (30 UI, by intraperitoneal route), 5 to 10 min before the administration of the anesthetic agent. The rats were then anesthetized with 80 mg/kg ketamine plus 10 mg/kg xylazine by intraperitoneal route. After obtaining deep anesthesia, a laparotomy was performed, allowing access to all the animal's viscera. The intestine was shifted to the right side, allowing visualization of the left kidney and dissection of the abdominal aorta. During these procedures, the viscera, especially the left kidney, were kept moist with surgical gauze soaked in physiological saline solution. With the aid of a surgical thread, the left ureter was ligated, preventing urinary flow and, consequently, the production of urine by the kidney. Another surgical wire was placed in the abdominal aorta artery, between the right and left renal arteries, to direct blood flow only to the left side. Blood flow in the aorta artery was interrupted below the right renal artery. Then, a small incision was made in the aorta artery, allowing the insertion of a catheter-directed to the left renal artery and fixed to the aorta artery with surgical threads. The surgical thread positioned over the abdominal aorta was tied, interrupting the renal flow, and a peristaltic perfusion pump was activated, starting the perfusion process. In the continuous perfusion process, the kidney was removed from the abdominal cavity and placed in a plate to remove the adjacent fat and adrenal gland, to avoid the influence of the adrenal on the renal vascular bed during the experimentation period. Then, the kidney was placed in a chamber containing 100 ml of physiological saline solution (PSS; 119 mM NaCl, 4.7 mM KCl, 2.4 mM CaCl_2_, 1.2 mM MgSO_4_, 25.0 mM NaHCO_3_, 1.2 mM KH_2_PO_4_, 11.1 mM dextrose, and 0.03 mM EDTA) and coupled to the perfusion system, kept at a temperature of 37°C, constantly aerated with 95% O_2_ and 5% CO_2_, with a constant flow of 4 ml/min. A period of 30 min was respected before starting the experimental evaluation. The recording of perfusion pressure was performed through a pressure transducer coupled to the perfusion system, connected to a computerized polygraph with specific integration software (PowerLab system and Chart 7.1 software, ADInstruments, Castle Hill, Australia). A diagram of the isolated perfused kidney experimental setup is displayed in Figure 1.

### 2.4. Evaluation of the Effects and Mechanisms of Boldine on the Renal Arteries

After a stabilization time of 30 min, tissue integrity was verified with a bolus injection of 120 mmol KCl. The integrity of the endothelium was checked 20 min later with the addition of phenylephrine (300 nmol) followed by acetylcholine (300 nmol). A further 20 min period waited, and then the preparations were continuously perfused with PSS containing phenylephrine (3 µM), which is enough to induce a sustained increase in renal perfusion pressure. After stabilization in the increase in perfusion pressure, the preparations received bolus injections containing 10, 30, 100, and 300 nmol of boldine, and the reduction in perfusion pressure was evaluated. The bolus injections of the tested substances were made in a final volume of 10 or 30 *μ*l through access close to the preparation, with a minimal time interval of 3 min between doses. Some kidney preparations were perfused with PSS that contained sodium deoxycholate (1.8 mg/ml) for 30 s to chemically remove the renal arteries' endothelium. After the infusion of sodium deoxycholate, regular PSS was perfused for 40 min for stabilization. The lack of vasodilatation confirmed the endothelium removal after a bolus injection of acetylcholine. A dose-response curve (30, 100, and 300 nmol) of boldine was generated for preparations without an endothelium. Responses were recorded 10 s after administration. Changes in perfusion pressure (in mmHg) were recorded and compared between groups.

In another experimental set, using preparations with an intact endothelium, the preparations were perfused with PSS that contained 3 *μ*M phenylephrine *plus* the following agents: 100 *μ*ML-NAME (nonselective nitric oxide synthase inhibitor), 1 *μ*M indomethacin (nonselective cyclooxygenase inhibitor), 40 mM KCl (K^+^-mediated depolarization), 10 mM TEA (nonspecific potassium channel blocker), 10 *μ*M glibenclamide (selective ATP-sensitive K^+^ channels blocker), apamin 10 nM (selective small-conductance Ca^2+^-activated K^+^ channel blocker), iberiotoxin (selective high-conductance Ca^2+^-activated K^+^ channel blocker), 1 nM charybdotoxin (selective high- and intermediate-conductance Ca^2+^-activated K^+^ channel blocker). After 15 min of perfusion, boldine (30, 100, and 300 nmol) was injected. Its ability to reduce perfusion pressure was compared with the results obtained with the control preparations perfused only with the vehicle.

### 2.5. Statistical Analysis

The results are expressed as the mean ± standard error of the mean from six preparations in each group. The statistical analyses were performed using a one-way analysis of variance (ANOVA) followed by Bonferroni's *post hoc* test. Values of *p* < 0.05 were considered statistically significant. The statistical analyses were performed using the GraphPad Prism software version 6.00 for Windows (GraphPad, La Jolla, CA, USA).

## 3. Results and Discussion

**Figure 2:**
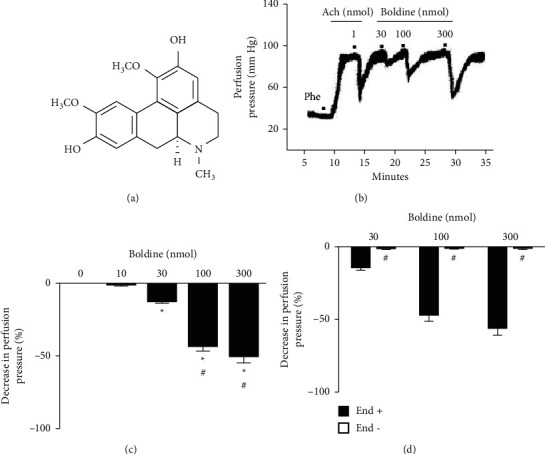
Boldine dose-dependently induced vasorelaxation in isolated and perfused kidneys. (a) Molecular structure of boldine. (b) Trace recording of kidney perfusion pressure showing the effects of acetylcholine (ACh) and boldine. (c) Effects of boldine on perfusion pressure in endothelium-intact isolated kidneys. (d) Effects of boldine on endothelium-intact (End+) and endothelium-denuded (End-) preparations. The data are expressed as the mean ± SEM from six experiments. ^*∗*^*p* < 0.05, compared with perfusion pressure after PSS administration (identified as the number 0); ^#^*p* < 0.05, compared with the previous dose of boldine (a) or endothelium-denuded preparations (b).

The vasodilator effect on renal arteries using isolated and perfused rat kidneys has been a heavily used strategy to assess the hemodynamic actions of isolated compounds, with a closer approximation to the mechanism of action, given the ability to assess different molecular targets [12]. In the present study, the administration of boldine (30, 100, and 300 nmol) in the vascular bed of the kidney induced a dose-dependent dilatory result (Figure 2(c)), thus answering the initial hypothesis of the work that discussed the possibility of boldine having a vasodilating action in the renal arterial bed. This is the first study to demonstrate the relaxing effects of boldine on the renal vascular bed. However, the relaxing effect of boldine on other vascular tissues was first described in 1996, where Chen et al. demonstrated the vasodilating action of this alkaloid in isolated rat thoracic aorta [13].

**Figure 3:**
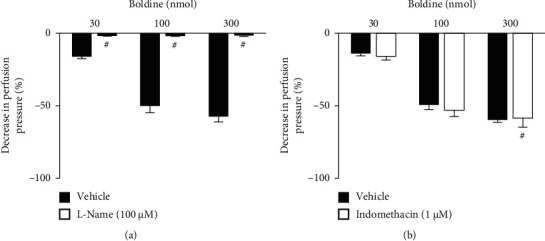
Role of nitric oxide and prostanoids in the vasodilatory effects of boldine. Effects of boldine on endothelium-intact kidney preparations that were perfused with (a) L-NAME or (b) indomethacin. The data are expressed as the mean ± SEM from six experiments. ^#^*p* < 0.05, compared with the vehicle-only perfused preparations.

Based on this positive result, investigations were carried out on the involvement of the endothelium and the underlying mechanisms related to relaxing actions. As depicted in Figure 2(d), the effects of boldine in the preparations previously administered with sodium deoxycholate (i.e., to remove the endothelium) were completely absent (Figure 2(d)), indicating that the functional endothelium is essential for its vasodilatory action. Correspondingly, the effects of all doses of boldine were prevented in preparations that were treated with the nonselective nitric oxide synthase inhibitor L-NAME (Figure 3(a)), while in the preparations perfused with the nonselective cyclooxygenase enzyme inhibitor indomethacin, the vasorelaxant effects of boldine remained unchanged (Figure 3(b)), indicating that endothelium-dependent relaxant properties of boldine were dependent of nitric oxide (NO) generation and independent of prostanoids production.

The main endothelium-derived relaxant factor is NO, which is considered one of the main mediators of cellular processes. In blood vessels, NO is produced in endothelial cells from L-arginine, having a vasodilating action. It diffuses to the smooth muscle cells interacting with the soluble guanylate cyclase enzyme making it active, causing the formation of cyclic guanosine monophosphate, which results in the relaxation of the vascular smooth muscle cell [14, 15]. Considering that endothelial removal or the suppression of NO production through the administration of L-NAME completely prevented the vascular effects of boldine, our findings suggest that the endothelium performed a crucial function in decreasing perfusion pressure induced by boldine. Indeed, the actions of boldine on vascular endothelium have already been described in the literature. Boldine treatment enhanced the maximal relaxation to acetylcholine in spontaneously hypertensive rats aorta [9], improved the endothelium-dependent relaxation in the aortas of streptozotocin-treated diabetic rats [16], and improved endothelium-dependent relaxation in aortas of diabetic mice [17].

**Figure 4:**
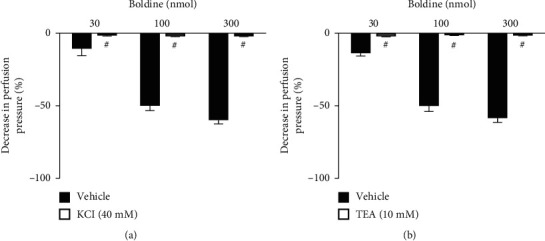
Effect of potassium channels on the vasodilatory effect of boldine. Effects of boldine on endothelium-intact kidney preparations that were perfused with (a) KCl or (b) TEA. The data are expressed as the mean ± SEM from six experiments. ^#^*p* < 0.05, compared with the vehicle-only perfused preparations.

**Figure 5:**
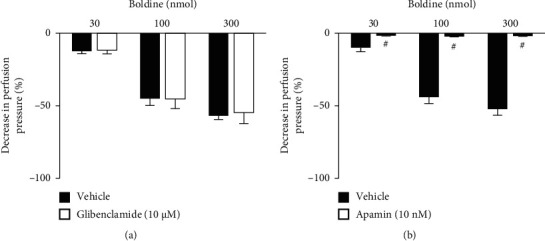
Effect of potassium channel subtype blockers on the vasodilatory effect of boldine. Effects of boldine on endothelium-intact kidney preparations that were perfused with (a) glibenclamide or (b) apamin. The data are expressed as the mean ± SEM from six experiments. ^#^*p* < 0.05, compared with the vehicle-only perfused preparations.

**Figure 6:**
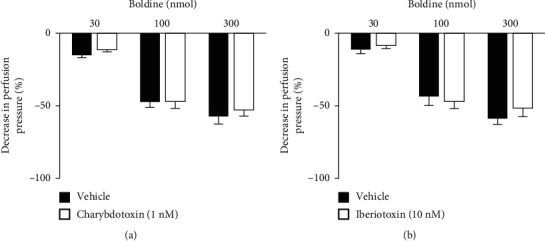
Effect of potassium channel subtype blockers on the vasodilatory effect of boldine. Effects of boldine on endothelium-intact kidney preparations that were perfused with (a) charybdotoxin or (b) iberiotoxin. The data are expressed as the mean ± SEM from six experiments.

To verify the actions related to K^+^ channels, the preparations were perfused with a nutrient solution in which 40 mM KCl was added, which induces K^+^-mediated depolarization, and TEA, a nonselective K^+^-channel blocker (Figures 4(a) and 4(b), respectively). It was observed that both treatments blocked the vasorelaxant effects induced by boldine, suggesting a predominant role of K^+^ channels in the vasodilatory effects of this compound. K^+^ channels are protein structures present in different types of cells, function as pores in membranes that allow the passage of K^+^. They are divided into 4 major classes: voltage-dependent K^+^ channels, ATP-sensitive K^+^ channels, inward-rectifier K^+^ channels, and Ca^2+^-activated K^+^ channels [18–20]. The treatment with glibenclamide, an ATP-sensitive K^+^ channel blocker, maintained the vasorelaxant action of boldine unchanged in all doses used (Figure 5(a)). Similarly, the perfusion with charybdotoxin, a selective high- and intermediate-conductance Ca^2+^-activated K^+^ channel blocker (Figure 6(a)), and iberiotoxin, a selective high-conductance Ca^2+^-activated K^+^ channel blocker (Figure 6(b)), did not modify the vasodilatory action of boldine. On the other hand, the perfusion with apamin, a selective small-conductance Ca^2+^-activated K^+^ channel blocker, completely prevented the vasodilatory action of boldine at all doses tested (Figure 5(b)), indicating that this K^+^ channel subtype appears to be crucial for the relaxing actions evoked by boldine. In summary, considering that the downstream targets of the nitric oxide pathway in vessels include the opening of K^+^ channels [21, 22], this set of results suggests that boldine-mediated endothelium-dependent vasodilation in renal arteries depends on the opening of small-conductance Ca^2+^-activated K^+^ channels induced by nitric oxide in smooth muscle cells.

It is noteworthy that, in recent years, many compounds isolated from medicinal plants have been studied about their renal actions, revealing important vasodilating, diuretic, antiurolithic, and protective actions against hypertensive damage. Nothofagin, a mono-C-glycoside of 4,2′,4′,6′-tetrahydroxy-dihydrochalcone found in *Leandra dasytricha* leaves, demonstrated the ability to induce diuresis [23], renoprotection [24], vasodilation [12], and hypotensive effect [25]. Different compounds from the xanthone classes isolated from *Garcinia achachairu* branches revealed acute diuretic actions [26, 27], prolonged diuretic and renoprotective effects [28], and antiurolithic properties [29]. Besides, glycosylated flavonoids obtained from the leaves of *Bauhinia forficata* revealed vasodilator [30], diuretic, and kidney protective effects [31].

However, although the results described herein undoubtedly point toward the participation of Ca^2+^-activated K^+^ channels in the renal effects induced by boldine, further studies are needed to investigate other molecular targets and subtypes of channels by using different inhibitors combined approaches. Besides, another important limitation of the study is that we were unable to explore the receptor involved in the vasodilatory effect of boldine, which remains to be studied in the future. Considering the actions already described in the literature for this alkaloid, this study adds about the renal vasodilator effect, contributing to a better understanding of boldine's potential to be used as pharmacotherapy or dietary supplement.

## Data Availability

The data used to support the findings of this study are available on request to the corresponding author.

## References

[1] Agra M. d. F., Freitas P. F. d., Barbosa-Filho J. M. (2007). Synopsis of the plants known as medicinal and poisonous in Northeast of Brazil. *Revista Brasileira de Farmacognosia*.

[2] Brandão M. G. L., Cosenza G. P., Moreira R. A., Monte-Mor R. L. M. (2006). Medicinal plants and other botanical products from the Brazilian Official Pharmacopoeia. *Rev Bras Farmacogn*.

[3] Viegas C., Bolzani V. S., Eliezer J., Barreiro E. J. (2006). The natural products and the modern medicinal chemistry. *Quimica Nova*.

[4] Ruiz A. L. T. G., Tafarello D., Souza V. H. S., Carvalho J. E. (2008). Pharmacology and toxicology of *Peumus boldus* and *Baccharis genistelloides*. *Rev. bras. Farmacognosia.*.

[5] O’Brien P., Carrasco-Pozo C., Speisky H. (2006). Boldine and its antioxidant or health-promoting properties. *Chemico-Biological Interactions*.

[6] Jang Y. Y., Song J. H., Shin Y. K., Han E. S., Lee C. S. (2000). Protective effect of boldine on oxidative mitochondrial damage in streptozotocin-induced diabetic rats. *Pharmacological Research*.

[7] Boeing T., Mariano L. N. B., Dos Santos A. C. (2020). Gastroprotective effect of the alkaloid boldine: involvement of non-protein sulfhydryl groups, prostanoids and reduction on oxidative stress. *Chemico-Biological Interactions*.

[8] Lau Y. S., Ling W. C., Murugan D., Mustafa M. R. (2015). Boldine ameliorates vascular oxidative stress and endothelial dysfunction. *Journal of Cardiovascular Pharmacology*.

[9] Lau Y.-S., Machha A., Achike F. I., Murugan D., Mustafa M. R. (2012). The aporphine alkaloid boldine improves endothelial function in spontaneously hypertensive rats. *Experimental Biology and Medicine*.

[10] Gómez G. I., Velarde V. (2018). Boldine improves kidney damage in the Goldblatt 2K1C model avoiding the increase in TGF-*β*. *International Journal of Molecular Sciences*.

[11] Hernández-Salinas R., Vielma A. Z., Arismendi M. N., Boric M. P., Sáez J. C., Velarde V. (2013). Boldine prevents renal alterations in diabetic rats. *Journal of Diabetes Research*.

[12] Marques A. A. M., da Silva C. H. F., de Souza P. (2020). Nitric oxide and Ca2+-activated high-conductance K+ channels mediate nothofagin-induced endothelium-dependent vasodilation in the perfused rat kidney. *Chemico-Biological Interactions*.

[13] Chen K. S., Ko F. N., Teng C. M., Wu Y. C. (1996). Antiplatelet and vasorelaxing actions of some aporphinoids. *Planta Medica*.

[14] Shiekh G. A., Ayub T., Khan S. N., Dar R., Andrabi K. I. (2011). Reduced nitrate level in individuals with hypertension and diabetes. *Journal of Cardiovascular Disease Research*.

[15] Underbakke E. S., Iavarone A. T., Chalmers M. J. (2014). Nitric oxide-induced conformational changes in soluble guanylate cyclase. *Structure*.

[16] Lau Y. S., Tian X. Y., Huang Y., Murugan D., Achike F. I., Mustafa M. R. (2013a). Boldine protects endothelial function in hyperglycemia-induced oxidative stress through an antioxidant mechanism. *Biochemical Pharmacology*.

[17] Lau Y. S., Tian X. Y., Mustafa M. R. (2013b). Boldine improves endothelial function in diabeticdb/dbmice through inhibition of angiotensin II-mediated BMP4-oxidative stress cascade. *British Journal of Pharmacology*.

[18] Baranowska M., Kozłowska H., Korbut A., Malinowska B. (2007). Potassium channels in blood vessels: their role in health and disease. *Postepy Higieny I Medycyny Doswladczalnej*.

[19] Jackson W. F. (2005). Potassium channels in the peripheral microcirculation. *Microcirculation*.

[20] Palmer B. F., Clegg D. J. (2016). Physiology and pathophysiology of potassium homeostasis. *Advances in Physiology Education*.

[21] Archer S. L., Huang J. M., Hampl V., Nelson D. P., Shultz P. J., Weir E. K. (1994). Nitric oxide and cGMP cause vasorelaxation by activation of a charybdotoxin-sensitive K channel by cGMP-dependent protein kinase. *Proceedings of the National Academy of Sciences*.

[22] Bolotina V. M., Najibi S., Palacino J. J., Pagano P. J., Cohen R. A. (1994). Nitric oxide directly activates calcium-dependent potassium channels in vascular smooth muscle. *Nature*.

[23] de Almeida C. L. B., Boeing T., Somensi L. B. (2017). Diuretic, natriuretic and potassium-sparing effect of nothofagin isolated from *Leandra dasytricha* (A. Gray) Cogn. leaves in normotensive and hypertensive rats. *Chemico-Biological Interactions*.

[24] de Almeida C. L. B., Cechinel-Filho V., Boeing T. (2018). Prolonged diuretic and saluretic effect of nothofagin isolated from *Leandra dasytricha* (A. Gray) Cogn. leaves in normotensive and hypertensive rats: role of antioxidant system and renal protection. *Chemico-Biological Interactions*.

[25] da Silva C. H., Palozi R. A., de Souza P. (2020). Nitric oxide/cGMP signaling pathway and potassium channels contribute to hypotensive effects of nothofagin. *Minerva Cardioangiologica*.

[26] Bolda Mariano L. N., Boeing T., da Silva R. d. C. M. V. d. A. F. (2019). 1,3,5,6-Tetrahydroxyxanthone, a natural xanthone, induces diuresis and saluresis in normotensive and hypertensive rats. *Chemico-Biological Interactions*.

[27] Bolda Mariano L. N., Boeing T., Cechinel-Filho V., Niero R., Mota da Silva L., de Souza P. (2020). The acute diuretic effects with low-doses of natural prenylated xanthones in rats. *European Journal of Pharmacology*.

[28] Bolda Mariano L. N., Boeing T., Cechinel Filho V. (2021). Prolonged diuretic and renoprotective effects of a xanthone obtained from *Garcinia achachairu* rusby in normotensive and hypertensive rats. *Evid-based Complement Alternative Medicine*.

[29] Mariano L. N. B., Boeing T., Cechinel Filho V., Niero R., Mota da Silva L., de Souza P. (2021). 1,3,5,6-tetrahydroxyxanthone promotes diuresis, renal protection and antiurolithic properties in normotensive and hypertensive rats. *Journal of Pharmacy and Pharmacology*.

[30] Cechinel-Zanchett C. C., da Silva R. d. C. M. V. d. A. F., Tenfen A. (2019). *Bauhinia forficata* link, a Brazilian medicinal plant traditionally used to treat cardiovascular disorders, exerts endothelium-dependent and independent vasorelaxation in thoracic aorta of normotensive and hypertensive rats. *Journal of Ethnopharmacology*.

[31] Cechinel-Zanchett C. C., Bolda Mariano L. N., Boeing T. (2020). Diuretic and renal protective effect of kaempferol 3-O-Alpha-l-rhamnoside (afzelin) in normotensive and hypertensive rats. *Journal of Natural Products*.

